# Mechanosensory neurons under pressure

**DOI:** 10.7554/eLife.96813

**Published:** 2024-03-13

**Authors:** Erin Cram

**Affiliations:** 1 Department of Biology, Northeastern University Boston United States

**Keywords:** neurons, neurodegenerative diseases, proteostress, mechanical stress, exophers, *C. elegans*

## Abstract

A build-up of eggs in the uterus of the nematode *C. elegans* triggers the release of large extracellular vesicles, called exophers, from neurons that are sensitive to mechanical forces.

**Related research article** Wang G, Guasp R, Salam S, Chuang E, Morera A, Smart AJ, Jimenez D, Shekhar S, Melentijevic I, Nguyen KC, Hall DH, Grant BD, Driscoll M. 2024. Mechanical force of uterine occupation enables large vesicle extrusion from proteostressed maternal neurons. *eLife* **13**:RP95443. doi: 10.7554/eLife.95443.

Being a neuron is stressful. Neurons tend not to be replaced in the body, so they need to remain healthy and able to adjust to changes in their environment and withstand a variety of chemical, electrical and mechanical challenges.

Keeping neurons healthy involves removing damaged proteins and replacing them with functional copies, and cells rely on a wide range of mechanisms to ensure that this happens (Redmann et al., 2016). However, damaged proteins can accumulate over time, and the build-up of such proteins, called proteostress, has been linked to Alzheimer’s disease and a number of other neurodegenerative diseases.

In the small nematode worm *C. elegans*, stressed neurons have been found to release large extracellular vesicles, known as exophers, which contain damaged organelles, large protein complexes and other protein aggregates (Melentijevic et al., 2017; Cocucci and Meldolesi, 2015, Cooper et al., 2021). However, the mechanism behind the extrusion of these exophers – which can measure up to 10 micrometers in diameter – remains unclear.

Now, in eLife, Monica Driscoll and colleagues – including Guoqiang Wang, Ryan Guasp and Sangeena Salam as joint first authors – report that *C. elegans* produces exophers in response to mechanical stress (Wang et al., 2024). The researchers – who are based at Rutgers, the State University of New Jersey and the Albert Einstein College of Medicine – studied neurons called lateral microtubule neurons, which are sensitive to touch and other mechanical forces. These neurons run along almost half of the length of the worm: the cell body is located near the uterus, and a long protrusion or neurite extends all the way to the head (Figure 1).

**Figure 1.**
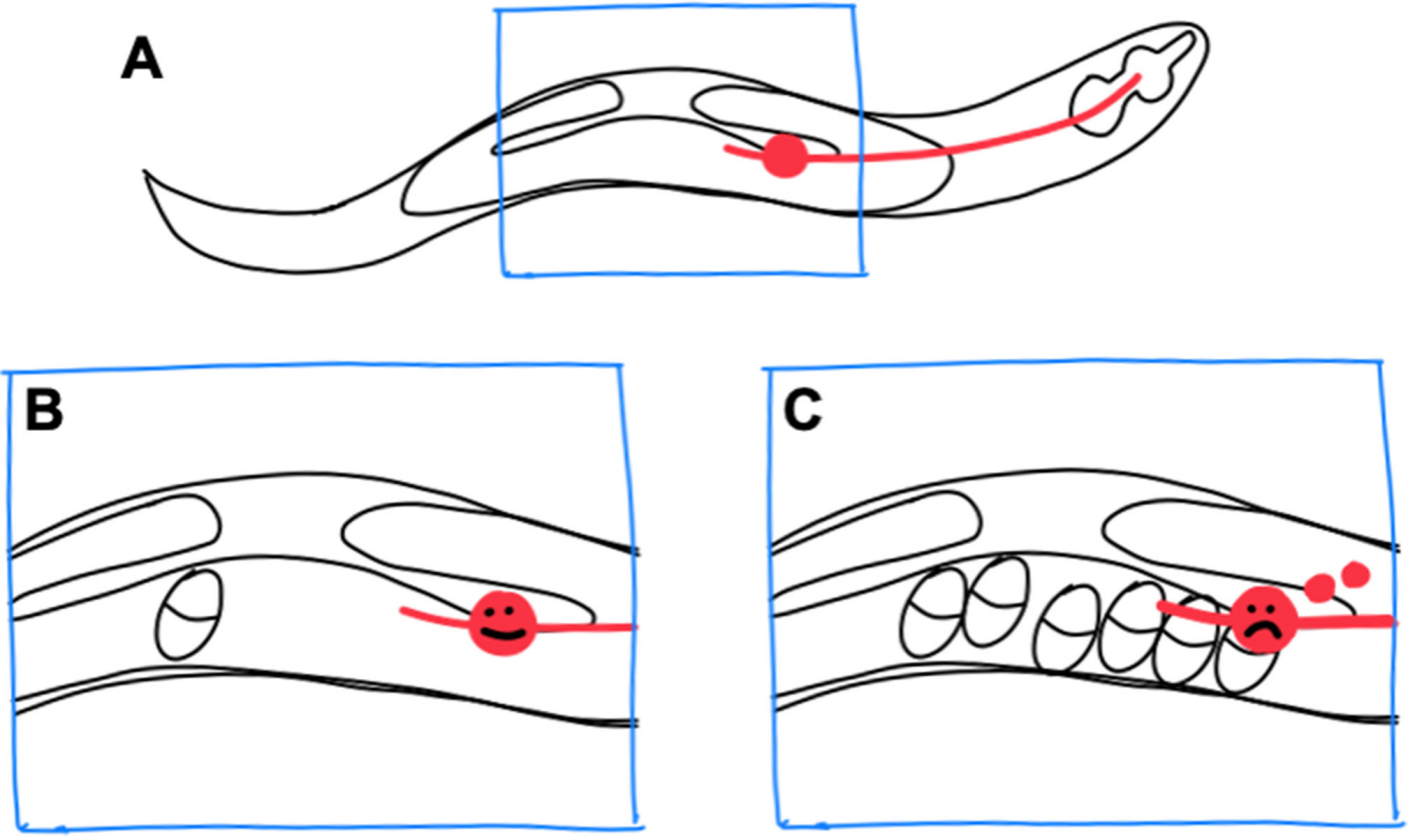
Exopher production by stressed neurons in *C*. *elegans*. (**A**) In the *C. elegans* hermaphrodite worm, the reproductive system consists of two gonad arms, which contain the germline, and a common uterus (indicated by the blue box), where eggs accumulate before they are laid. The cell body of a lateral microtubule neuron (red circle) is in close proximity to the uterus, and the neurite of this neuron (red line) extends all the way to the head of the worm. (**B**) When only a small number of eggs are in the uterus, the neuron does not produce exophers. (**C**) However, when eggs accumulate in the uterus, the cell body becomes stressed and exophers (small red circles) are released.

Wang et al. analyzed the production of exophers in neurons expressing a fluorescent protein known as mCherry. High levels of expression of mCherry lead to proteostress in the neurons and also made it possible to trace the production and extrusion of exophers.

The experiments revealed that the release of the exophers varied with time, reaching a peak when the number of eggs in the uterus was at its highest. Using a variety of different genetic manipulations, Wang et al. showed that blocking the production of eggs inhibited the production of exophers, while increasing the load of eggs in the uterus promoted exopher production.

Moreover, the researchers found that the position of the neurons relative to the eggs mattered, and that neurons with cell bodies in the ‘egg zone’ produced more exophers. Likewise, in mutant worms with an expanded egg zone, neurons in the general vicinity of the zone produced large numbers of exophers, whereas those outside the zone did not.

Eggs distort the uterus and press into the surrounding tissues, so it is possible that the production of exophers is triggered by the physical presence of the eggs, rather than by chemical signals released by them. Wang et al. showed that filling the uterus with anything – dead eggs, unfertilized oocytes, or a buffer solution – leads to the production of exophers, which suggests that this process is triggered by the physical presence of the eggs.

Many intriguing questions remain. For example, how do neurons sense the mechanical signal, and how exactly does mechanical stress lead to the production of exophers? Wang et al. suggest that exopher production could be mediated through mechanosensitive ion channels such as PEZO-1/Peizo (Delmas et al., 2022). This ion channel is expressed in many tissues in *C. elegans*, including the lateral microtubule neurons, and an influx of calcium ions through it could stimulate the release of exophers. Alternatively, mechanical information could be transmitted via the extracellular matrix to integrins or other receptor proteins on the surface of the neuron. Moreover, the nuclei of some neurons are able to sense pressure (Niethammer, 2021), and a role for the nuclei in sensing mechanical forces would be consistent with the fact that exopher production is influenced by the position of the cell body, rather than the position of the neurites.

It remains to be seen if exophers are merely responsible for waste disposal, or if they also transmit products and/or information between cells. For example, *C. elegans* embryos can stimulate muscle cells to release exophers that are full of yolk: these exophers are then taken up by oocytes, and the yolk is used as food by the developing embryos (Turek et al., 2021). Exophers released from neurons can be also taken up by other cells and may potentially convey information about the stressed state of the worm to these other cells (Melentijevic et al., 2017; Turek et al., 2021; Wang et al., 2023).

Cell-maintenance systems, such as autophagy, are important for the survival of organisms, but the production of exophers may provide an important backup. For example, it was shown recently that exopher production can extend lifespan when autophagy is blocked in *C. elegans* neurons (Yang et al., 2024). A better understanding of the conserved mechanisms of exopher production and function may help reveal how neurons adapt and survive under stressful conditions, and further elucidate the full potential of exophers.
